# Enhancing Patient Safety Through the Prevention of Healthcare Facility-Onset Methicillin-Resistant Staphylococcus aureus Bacteremia

**DOI:** 10.1017/ash.2025.376

**Published:** 2025-09-24

**Authors:** Taylor Langston, Jessica Beltran, Ioana Chirca, Alexis Marano, Allison Mott

**Affiliations:** 1 AdventHealth; 2 AdventHealth Orlando

## Abstract

**Background:** Healthcare facility-onset (HO) Methicillin-Resistant Staphylococcus aureus (MRSA) bacteremia represents a critical patient safety issue due to its associated morbidity and mortality. This metric is reportable to the Centers for Medicare and Medicaid Services (CMS) and impacts Leapfrog scores. However, the primary motivation for this investigation was to enhance patient safety by addressing opportunities for improvement identified through reporting trends. This project sought to identify root causes and implement targeted quality improvement measures to strengthen HO MRSA bacteremia prevention efforts. **Method:** A standardized root cause analysis (RCA) template was developed and applied across a tri-county region with nine acute care hospitals. The template included 58 variables based on local and national MRSA prevention measures and National Healthcare Safety Network (NHSN) recommendations. Cases meeting NHSN HO MRSA criteria were included in the analysis. Infection preventionists reviewed electronic medical records and documented findings using the RCA template. Abstracted data were analyzed by the hospital epidemiologist using Excel QI macros. The RCA findings highlighted gaps in peripheral intravenous line (PIV) care and maintenance as potential contributors to bloodstream infection risk. Building on these findings, a targeted nursing survey was conducted to identify barriers, gaps, and opportunities for improvement in PIV care practices, further informing the development of actionable quality improvement interventions. **Result:** Thirty-two HO MRSA cases were reported in 2023, 38.9% of which involved blood cultures with no other identified source. Specimen collection dates clustered around line days 4-10 and 18-22. Eighteen cases documented catheter-related issues, primarily drainage and infiltration at PIV sites. The RCA findings prompted a nursing survey to investigate PIV care practices. Survey responses revealed inconsistencies in maintenance practice, variation in documentation and monitoring, and requests for increased training and refresher sessions. These insights validated the RCA findings and informed the design of targeted interventions aimed at improving PIV care. **Conclusion:** HO MRSA bacteremia prevention requires a comprehensive approach that prioritizes patient safety while leveraging RCA findings and frontline staff input. The RCA findings and nursing survey have provided critical insights into gaps in PIV care and maintenance, laying the foundation for actionable quality improvement measures. Planned interventions, including re-education on PIV best practices, addressing survey-identified barriers, and implementing a bloodstream infection prevention bundle, are set for implementation in the next calendar year. These efforts aim to strengthen HO MRSA bacteremia prevention and improve patient outcomes.

